# A Strategy for Accessing Nanobody-Based Electrochemical Sensors for Analyte Detection in Complex Media

**DOI:** 10.1149/2754-2726/ac5b2e

**Published:** 2022-04-07

**Authors:** Ruolan Fan, Yanfeng Li, Kwang-Won Park, Jiale Du, Lin Hui Chang, Eric R. Strieter, Trisha L. Andrew

**Affiliations:** 1 Department of Chemistry, University of Massachusetts Amherst, Amherst, Massachusetts, United States of America; 2 Department of Chemical Engineering, University of Massachusetts Amherst, Amherst, Massachusetts, United States of America

## Abstract

Nanobodies are single variable domain antibodies isolated from camelids and are rapidly distinguishing themselves as ideal recognition elements in biosensors due to their comparative stability, ease of production and isolation, and high binding affinities. However, transducing analyte binding by nanobodies in real time is challenging, as most nanobodies do not directly produce an optical or electrical signal upon target recognition. Here, we report a general strategy to fabricate sensitive and selective electrochemical sensors incorporating nanobodies for detecting target analytes in heterogeneous media, such as cell lysate. Graphite felt can be covalently functionalized with recombinant HaloTag-modified nanobodies. Subsequent encapsulation with a thin layer of a hydrogel using a vapor deposition process affords encapsulated electrodes that directly display a decrease in current upon antigen binding, without added redox mediators. Differential pulse voltammetry affords clear and consistent decreases in electrode current across multiple electrode samples for specific antigen concentrations. The change in observed current vs increasing antigen concentration follows Langmuir binding characteristics, as expected. Importantly, selective and repeatable target binding in unpurified cell lysate is only demonstrated by the encapsulated electrode, with an antigen detection limit of ca. 30 pmol, whereas bare electrodes lacking encapsulation produce numerous false positive signals in control experiments.

Recent reports of wearable and/or flexible electrochemical biosensors are centered around small-molecule biomarkers, such as glucose,^1,2^ lactose,^3,4^ and uric acid,^5–7^ while comparatively few research endeavors^8–10^ are focused on broadening the current point-of-care map from small molecules to biomacromolecules, such as proteins or peptides. To non-exhaustively highlight a few notable reports: Janegitz et al. fabricated flexible platinum electrodes on biobased poly(ethylene terephthalate) (Bio-PET) to detect Parkinson’s disease protein 7(PARK7/DJ-1) by electrochemical impedance spectroscopy;^11^ Yadavalli et al. created electrodes on flexible fibroin substrates to target the vascular endothelial growth factor (VEGF), which is a critical indicator for wound healing or early disease diagnosis;^12^ and Tiwari et al. used functionalized, flexible aluminum foil substrates for simultaneously detecting the diabetes indicators glycated hemoglobin and non-glycated haemoglobin.^13^ However, the electrode fabrication process for most macromolecular targets remain specific to the biorecognition element used and cannot be readily translated to create sensors for a broad range of targets. Furthermore, antibodies, which are the most common bioreceptors for protein detection, are not always stable upon immobilization onto flexible electrodes, and can decompose or lose their function unless stored or handled under stringent conditions. Lastly, the cost and timeline for producing, isolating and purifying antibodies is significant and, therefore, biosensors containing antibody recognition elements are comparatively expensive.

Single variable domain (VHH) antibodies from camelids, which are called nanobodies, have attracted a great deal of attention as recognition elements for next-generation biosensors.^14,15^ Analogous to their more well-known and widely used counterparts, immunoglobulin G (IgG) antibodies, nanobodies are capable of binding antigens with high affinity and specificity.^16–18^ What sets nanobodies apart from IgGs is their smaller size (12–15 kDa as opposed to ca.150 kDa), high thermal stability, wide pH tolerance, and ease of recombinant production. Due to these advantageous characteristics, nanobodies can enable next-generation sensing platforms that retain their recognition ability upon exposure to harsh or variable conditions and are also capable of directly and selectively binding antigens in complex matrices, such as cell lysate, without the need for added purification or preconcentration steps.

However, to date, real-time optical and/or electrochemical transduction of antigen binding has not been demonstrated for nanobody-based sensing elements. Most known nanobodies do not produce a direct optical or electrical signal concomitant with target recognition and, therefore, various co-reagents, redox mediators, or specially synthesized fluorophore-functionalized antigens^19,20^ or fluorogens^21,22^ must be added to affect an observable signal. While this general process enables several powerful biochemical assays to be performed in a lab, multicomponent analysis mixtures are not ideal for portable, point-of-care sensing systems. Another important consideration is that site-specific immobilization is necessary when working with nanobodies to ensure that their target recognition capability can be preserved upon immobilization onto a solid support/surface–nonspecific immobilization effected by commonplace peptide coupling or thiol-addition chemistries can often deactivate or attenuate the specificity and sensitivity of nanobodies.

Here, we report a straight-forward process to extract a direct electrochemical response from nanobody-decorated conductive soft substrates. Briefly, nanobodies were first site-specifically immobilized on functionalized graphite felt through HaloTag chemistry^23^ and encapsulated with a thin, biocompatible hydrogel layer—poly(2-hydroxyethyl methacrylate) (PHEMA)^24,25^—through photoinitiated chemical vapor deposition (piCVD). We previously demonstrated that recombinantly expressed nanobodies, which contain the functional HaloTag moiety at an innocuous site, maintain their target recognition capability upon immobilization. The graphite felt-based sensor described herein presented robust, quantitative performance towards the purified target antigen and, also, demonstrated high specificity when treated with the same target antigen in complex, heterogeneous mixtures, such as unpurified cell lysate. Besides notable sensitivity, the fully-encapsulated sensors also demonstrated high stability and repeatability compared to unencapsulated or bare sensors. The nanobody chosen for these studies, NbIII.15, was derived from a synthetic yeast surface display library and engineered to bind with high affinity and specificity to the human protein UCHL5/UCH37. This nanobody was chosen for this initial proof-of concept due to availability in our labs, but the procedures we report can be easily applied to any known nanobody-antigen combination or used to develop nanobody-based electrochemical biosensors for any biomolecule or target of interest.

## Experimental

### Materials and reagents.—

Potassium ferrocyanide, potassium ferricyanide, (3-aminopropyl)trimethoxysilane (APTMS), 6-chlorohexanoic acid (CHA), N-(3-dimethylaminopropyl)-N’-ethylcarbodiimide hydrochloride (EDC), dichloromethane (DCM), ethanol, 2-hydroxy-2-methylpropiophenone (HMPP), 2-hydroxyethyl methacrylate (HEMA) and sulfuric acid were purchased from Millipore-Sigma and were used without further purification. Graphite felt (GF) of 2 mm thickness was purchased from the Fuel Cell Store.

### Substrate preparation.—

Graphite felt was cut into 1.5 cm × 0.5 cm pieces and electrochemically activated in 0.1 M sulfuric acid by sweeping from −2500 mV to 2500 mV for 3 cycles at 100 mV s ^−1^ in a three-electrode system (Ag/AgCl as reference electrode and Pt wire as counter electrode). After rinsing with DI water and drying under air, activated graphite felt was immersed in a 2% APTMS solution in ethanol/water (90/10 v/v %) for 2 h. Then the felt was washed with pure ethanol three times and blow-dried with forced air. To introduce the chloroalkane group, the substrates were treated with 5 ml DCM containing 0.01 mmol EDC and 0.01 mmol CHA for 12 h. The grafted samples were further washed with pure DCM three times, dried with forced nitrogen and used immediately for Nb immobilization.

### Protein expression and purification.—

Protein expression and purification were discussed in our previous study.^26^ Briefly speaking, halo-tagged NbIII.15 constructs were expressed in BL21 (DE3) pLysS Escherichia coli cells in LB media supplemented with kanamycin (25 *μ*g ml^−1^) at 37 °C to OD600 ~ 1.0 and induced with 300 μM IPTG at 16 °C for 16 h. The clarified lysate was obtained from cell pellets resuspended in lysis buffer (50 mM Tris-HCl pH 8.0, 300 mM NaCl, and 1 mM TCEP) after sonication and centrifugation, incubated with Ni-NTA resin for 2 h at 4 °C, washed with lysis buffer, and eluted with lysis buffer plus 300 mM imidazole. The eluate was then buffer-exchanged into gel filtration buffer (50 mM Tris-HCl pH 8.0, 300 mM NaCl, and 1 mM DTT) and ran on a Superdex 75 (GE) gel filtration column at 0.3 ml min^−1^.

### Cell lysate generation.—

Wild-type (UCH37) and UCH37 knockout (UCH37^KO^) HEK293 cells stably expressing RPN11-HTBH were grown, harvested, and lysed in the lysis buffer (40 mM HEPES pH 7.4, 40 mM NaCl, 10 mM MgCl2, 2 mM ATP, 1 mM DTT, and 10% glycerol).^27^ The HEK293 lysates were clarified at 20,000xg for 20 min and the supernatant was collected and then stored at −80 °C prior to use. The concentration of total cell lysate was determined by bicinchoninic acid assay.

### Nb immobilization.—

CHA functionalized graphite felt substrates were first soaked in phosphate-buffer saline (PBS) for 5 min at room temperature. Three 1.5 cm × 0.5 cm pieces were immersed in approximately 5 ml of PBS in a petri dish. Halo-tagged control Nb or NbIII.15 were diluted to 5 *μ*M in PBS and added to the graphite felt substrates in PBS. These samples were then incubated at 4 °C overnight with rocking. Then, the felt was washed with PBS three times for 5 min to remove the unreacted Nb and dried under air.

### Polymer encapsulation.—

Polymerization of HEMA through photoinitiated chemical vapor deposition (piCVD) was conducted in a custom-built reactor (stainless-steel walls, 290 mm diameter, and 70 mm height) with a low-intensity UV-lamp (UVP, UVLS-24 EL Series, 4 W, 254 nm). During deposition, the base pressure of the reactor was kept at 200 mTorr, while the stage temperature was maintained at 20 °C with a recirculating cooling system. Photoinitiator HMPP and monomer HEMA were heated to 110 °C in separate glass ampules wrapped with fiberglass heating tape and introduced into the reactor through articulated needle valves. The polymerization/deposition was allowed to proceed for 5 min with the needle valves open and UV light source turned on and followed by a 30 min vacuum annealing step (ampule heating tapes and UV light turned off, needle valves closed, recirculating cooling system on) to pull out unreacted monomers and photoinitiators from film created on the graphite felt samples.

### Electrochemical characterization.—

Electrochemical measurements, including differential pulse voltammetry (DPV), were carried out using a WaveNow potentiostat from Pine Instruments. All experiments were conducted using a three-electrode setup, with a standard Ag/AgCl reference electrode and a platinum wire counter electrode. For each data point, current at 0.1 V was collected and at least three parallel experiments were performed to ensure repeatability. During DPV measurements, the increment (interval) voltage in pulse parameters was set as 10 mV, with height of 50 mV and width of 20 ms to ensure reliable data collection. The redox mediator solution used for quantifying Nb binding events was composed of 5 mM potassium ferricyanide and 5 mM ferrocyanide in PBS. Impedance measurements were performed using a Solartron Analytical SI 1287 Electrochemical Interface with a 1252 A Frequency response analyzer. Samples were tested over the frequency range from 300 000 to 0.1 Hz at an AC amplitude of 50 mV. The resulting Nyquist plots were modeled and fitted using the ZView software (Scribner Associates).

## Results and Discussion

Carbon materials are commonly used as electrodes due to their high chemical stability in various media and high electrical conductivity. Over decades, advancements in electrode modification have been achieved by the introduction of the graphene, carbon nanotubes and other nanomaterials with specific functions.^28^ However, one weakness is that the close-packed nature of thin films of these carbon nanomaterials translates to reduced electrode surface area. Graphite felt possess the general benefits of modified carbon electrodes while also boasting porosity and large surface area due to its three-dimensional fibrous assembly, which allows for effective mass transfer and charge transfer across the active surface area. Graphite felt also displays enhanced mechanical integrity,^29^ which particularly qualifies it as a preferred substrate for bio-relevant, wearable applications.

**Figure 1.**
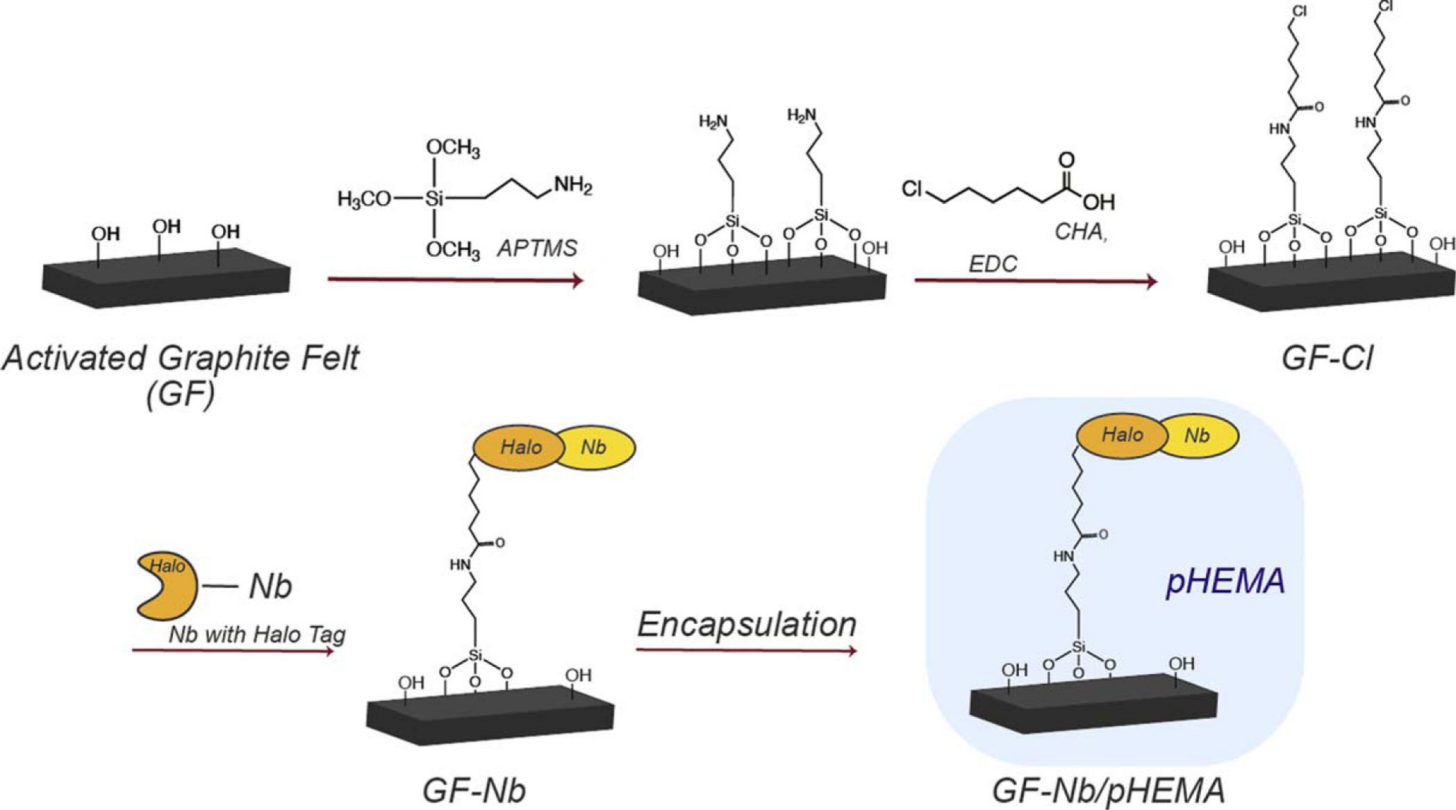
Summary of the process to create nanobody-decorated, encapsulated graphite felt working electrodes.

**Figure 2.**
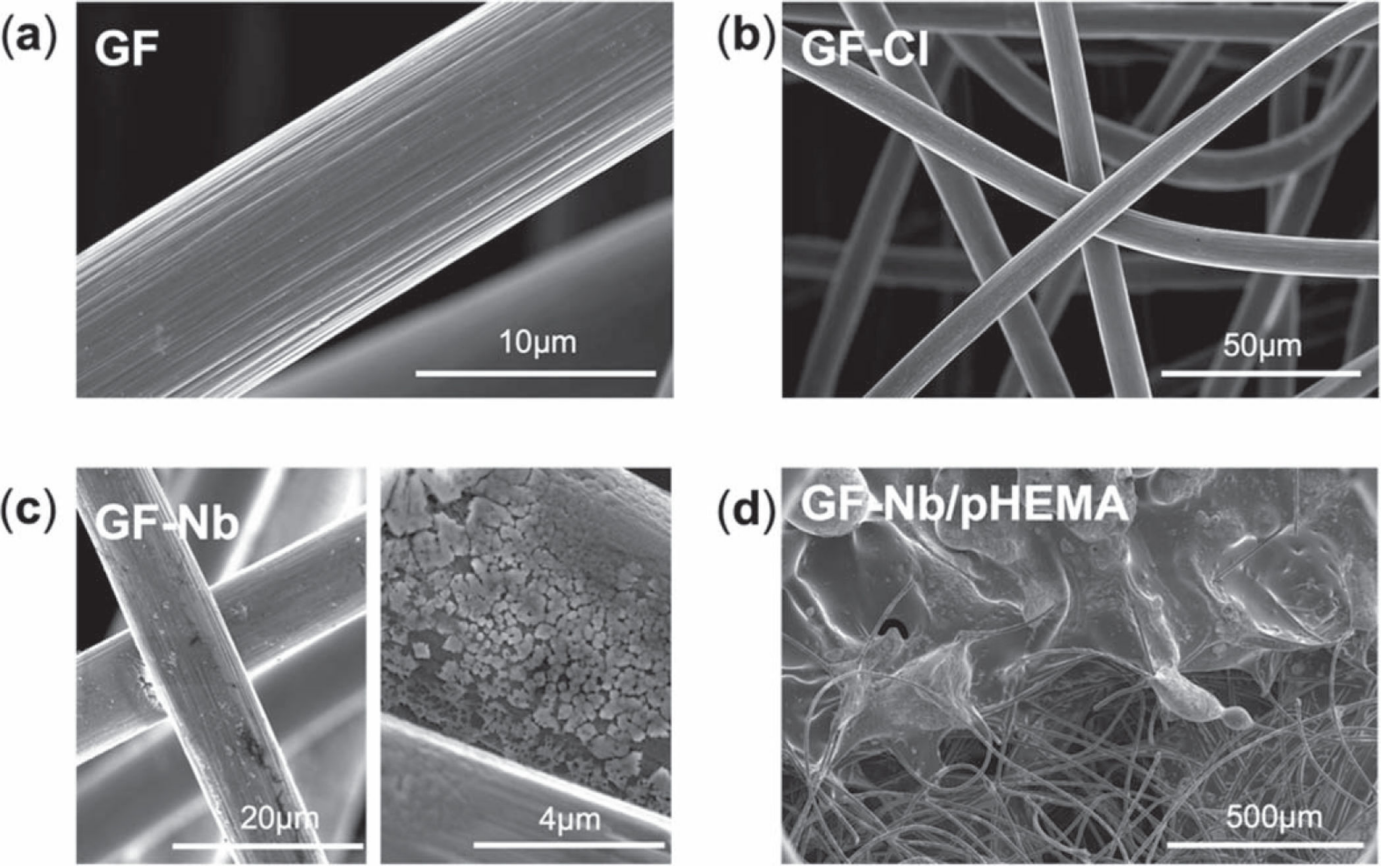
Scanning electron micrographs of (a) pristine graphite felt (GF), (b) graphite felt after silanization with APTMS and subsequent reaction with CHA (GF-Cl), (c) graphite felt covalently decorated HaloTag-recombinant Nb (GF-Nb), and (d) the nanobody-functionalized graphite felt after encapsulation with PHEMA.

The process used to create Nb-functionalized working electrodes is summarized in Fig. 1. Surface silanization of electrochemically-activated graphite felt using APTMS enabled the introduction of amine functional groups, which were subsequently coupled to 6-chlorohexanoic acid (CHA) to afford a HaloTag-reactive surface. The functionalized graphite felt was then incubated with an engineered version of nanobody NbIII.15 containing a HaloTag protein tag to effect nanobody immobilization. Scanning electron microscope (SEM) images of the functionalized GF electrodes are shown in Fig. 2. Changes in the macroscale texture and the average nanoscale fiber diameter of the graphite felt were not observed after the APTMS/CHA reaction sequence, as expected, confirming that this surface functionalization sequence did not create unwanted polymer coatings or otherwise corrode the felt. Energy-dispersive Xray (EDX) analysis of the graphite felt after the APTMS/CHA reaction revealed the presence of Si (as nitrogen cannot be easily detected in SEM, silicon was chosen instead) and Cl atoms on the surface (Fig. S1 (available online at stacks.iop.org/ECSSP/1/010601/mmedia)), even after multiple rinsing steps, indicating that the silanization and amidation reactions proceeded as expected. SEM images of the GF after incubation with HaloTag-Nb revealed significant changes in the nanoscale texture of the surface of the graphite felt and additional layers were observed wrapping around the fibers, suggesting that the bioreceptors were successfully introduced onto the surface of functionalized graphite felt. Large area images revealed that the nanobody-functionalized graphite felt samples had uniform textures and fiber morphology over at least 1 micron, suggesting a relatively uniform distribution of the bioreceptors throughout the felt.

**Figure 3.**
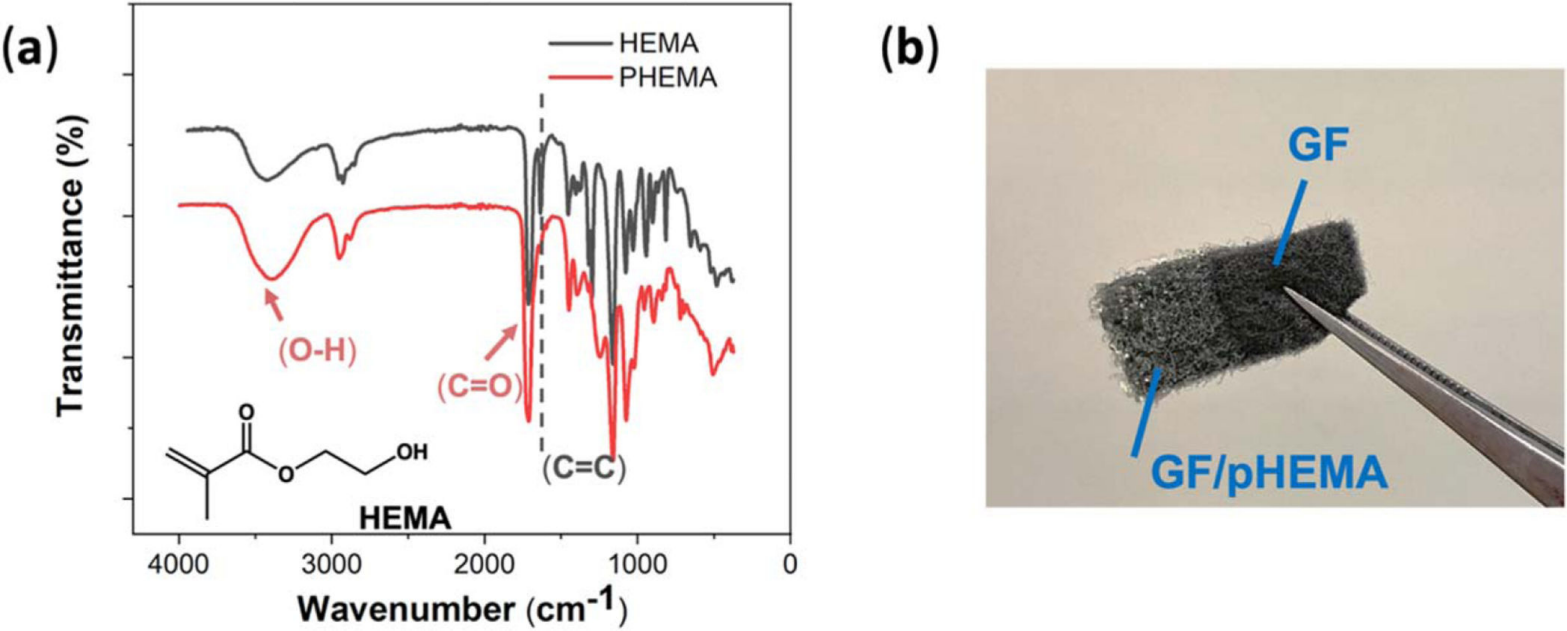
(a) Fourier transform IR spectrum of the monomer HEMA and the polymer film/coating, PHEMA, that is formed after the piCVD process, showing disappearance of the acrylic double bond upon polymerization. (b) Optical image of a partially functionalized and encapsulated graphite felt sensor.

Next, the nanobody-functionalized graphite felt was encapsulated to minimize false positive signals from nonspecific binding and/or interferent adsorption onto the sensor surface.^26^ To prevent the Nb from being exposed to organic solvents and to conformally, uniformly coat the disordered three-dimensional fibrous network of the graphite felt electrodes, photoinitiated chemical vapor deposition (piCVD) was used to deposit a thin encapsulation layer. We also note that graphite felt acts much like a sponge for many solvents and, therefore, a solution-based polymer deposition process would likely significantly disrupt the weight, volume, packing structure and fiber network of the graphite felt, thus further recommending a solvent-free vapor deposition process for encapsulating graphite felt-based electrodes. For this study, a hydrogel coating (PHEMA) was applied to the nanobody-functionalized graphite felt because this polymer was experimentally found to produce uniform coatings over large (cm-scale) areas of the graphite felt whereas other, previously-reported polymers yielded nonuniform coatings with undesirable polymer agglomeration. Figure 2d shows an SEM image of the boundary between a PHEMA-encapsulated and unencapsulated region of a nanobody-functionalized graphite felt electrode; the top half of the image shows the PHEMA encapsulation layer. An optical image of a nanobody-functionalized graphite felt electrode that is partially encapsulated with PHEMA is provided in Fig. 3b. The FTIR image of the PHEMA encapsulation layer is provided in Fig. 3a and is compared to the FTIR spectrum of the monomer, HEMA. The major IR absorption bands of PHEMA are due to the stretching vibration of C=O at 1720 cm^−1^ and −OH at 3200–3600 cm^−1^.^30^ Noticeably, although the overall piCVD run time was less than 5 min, the stretching peak of the unsaturated C=C bonds at 1630 cm^−1^ seen in the monomer FTIR spectrum was not present in the PHEMA spectrum, supporting the high polymerization efficiency of the piCVD process.

The electrochemical characteristics of the graphite felt electrode at various levels of functionalization were investigated. Graphite felt is a disordered three-dimensional fibrous network substrate with high surface area, which means that a large background signal will necessarily be generated when performing sensitive electrochemical measurements. As a result, all electrochemical signals generated by a bare graphite felt electrode were observed to be noisier and less well-defined as compared to the signals afforded by metal-based electrodes. To account for this intrinsic substrate variation, for each measurement described herein, experimental parameters were fixed and at least three parallel experiments were performed to ensure repeatability.

**Figure 4.**
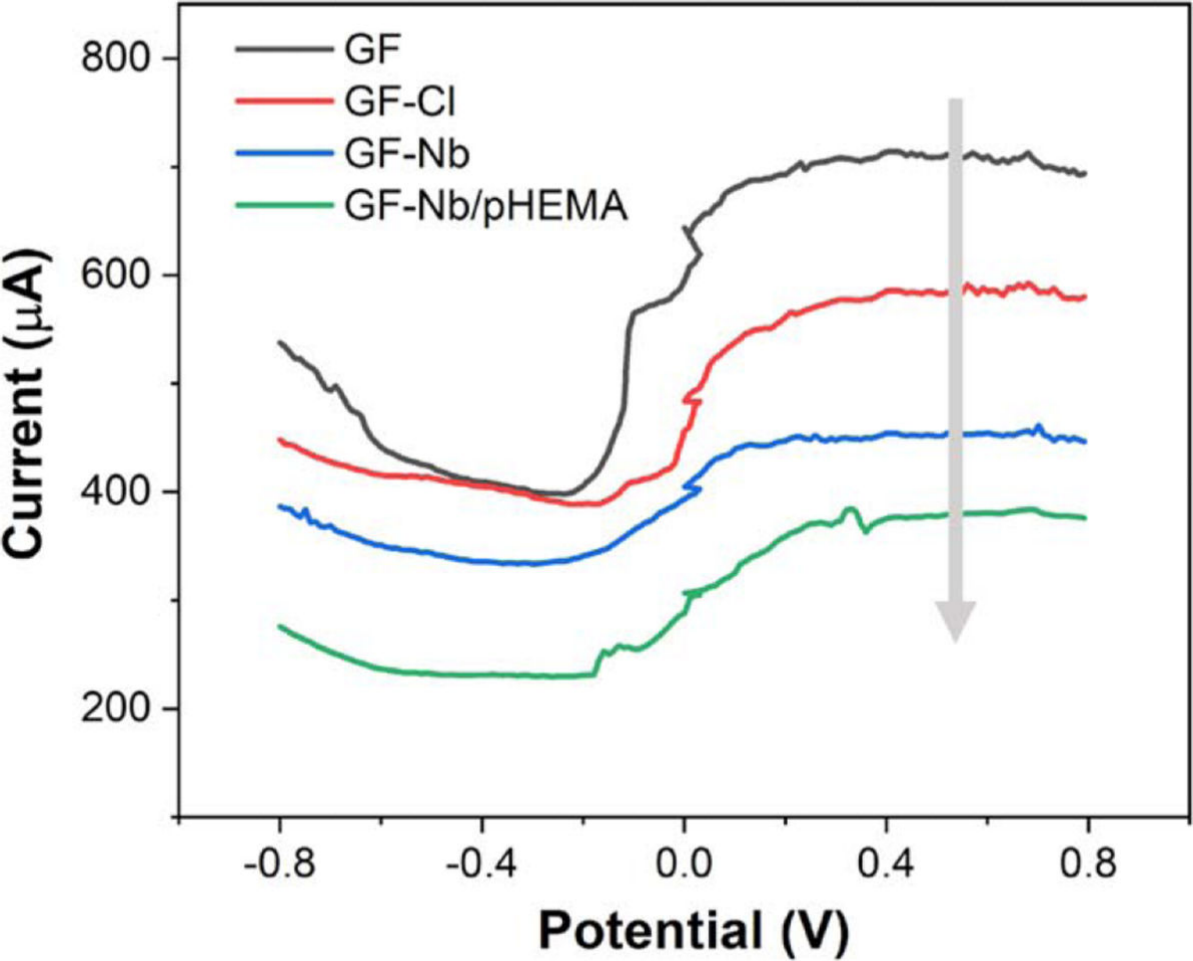
Observed current signals from differential pulse voltammetry performed using graphite felt working electrodes after each level of surface functionalization.

Differential Pulse Voltammetry (DPV) measurements were selected to quantify current changes caused by surface functionalization while excluding any non-faradaic currents induced by the dense fiber network of graphite felt. Generally, when performing electrochemical measurements, the current generated from surface-functionalized working electrodes in solution tends to decrease when the complexity of the surface functionalization increases (assuming the functional coatings contain a majority of insulating or nonconductive components that hinder mass/charge transfer to the electrode).^31^ This expected trend is indeed observed for the functionalized graphite felt electrodes created here, at each step of our functionalization process. As seen in Fig. 4, starting from bare electrochemically-activated graphite felt, the apparent current signal afforded by DPV steadily decreases as the electrode is silanized, then functionalized with a nanobody recognition element and encapsulated with a PHEMA hydrogel (all insulating components). The surface modifications, like Nb immobilization and encapsulation, helped to smooth out the “fuzzy,” disordered surface morphology of bare graphite felt and, thus, lead to reduced background noise for subsequent sensing applications.

Impedance spectra of two electrode systems are shown in Fig. S2. The charge transfer process between redox mediators in the system and the electrodes, although attenuated with increasingly complex surface functionalization, was never completely absent in the functionalized and encapsulated electrode, thus confirming that the surface functionalization protocols described herein effectively produce working electrodes for electrochemical sensing. Notably, however, impedance spectroscopy revealed that the encapsulation layer must be thin (approximately 300 ± 200 nm) to produce functional working electrodes. In our fabrication protocols, we limited the piCVD run time for the hydrogel encapsulation layer to ensure a relatively thin encapsulation layer on the electrode and protect the Nb from degradation. This short run time produced a thin PHEMA film on the electrode (an observed thickness of 200 nm on a silicon wafer coupon), which minimized the insulating effect of a polymer encapsulation layer on the working electrode. As illustrated in Fig. S3(a), longer deposition times (15 min) resulted in thicker (> 1 micron) encapsulation layers and widespread polymer agglomeration on the fiber networks within the graphite felt, which weakened the charge transfer process between the electrolyte and encapsulated electrode and resulted in a flatter current curve near 0.1 V (Fig. S3b).

**Figure 5.**
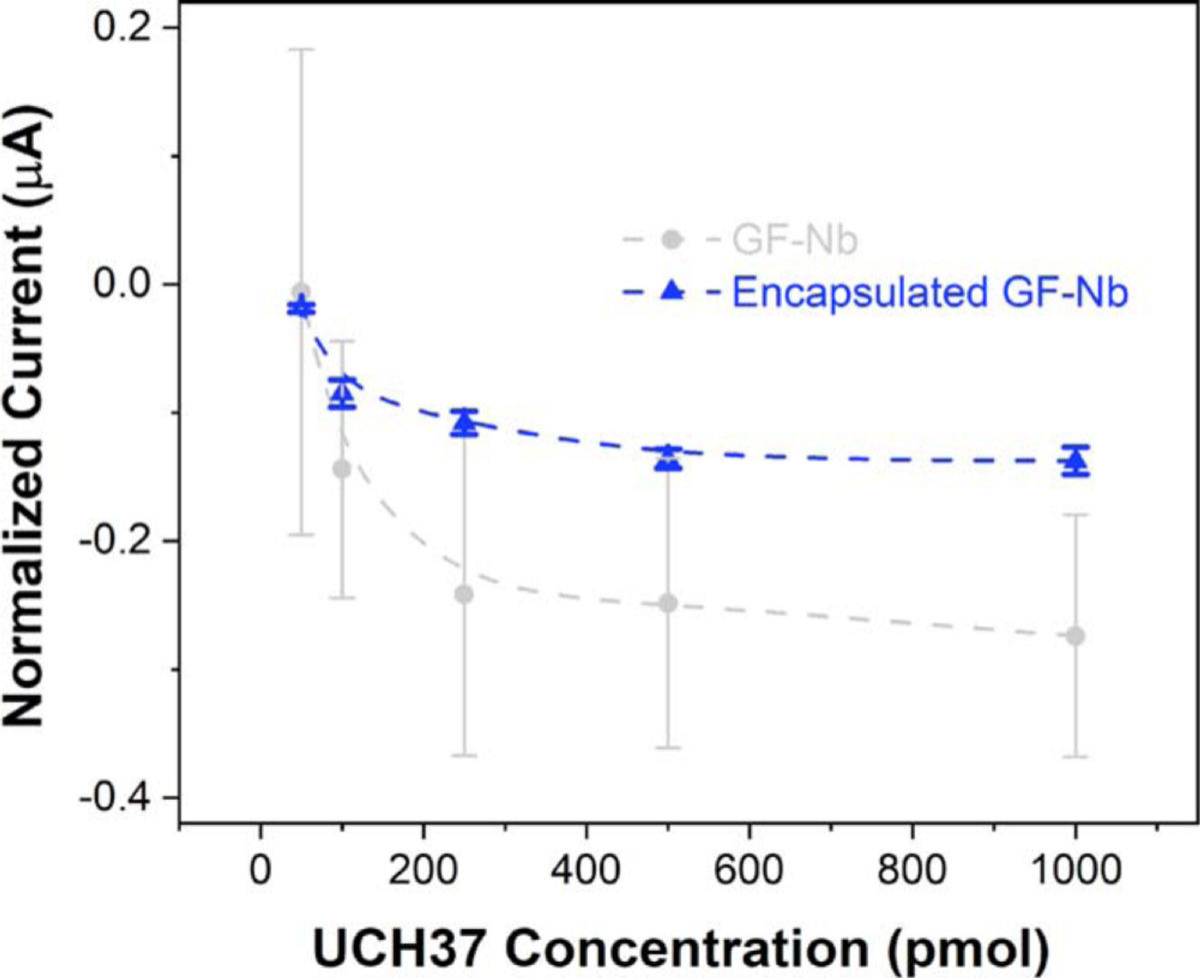
Observed current signals from differential pulse voltammetry performed using bare GF-Nb electrodes and encapsulated GF-Nb electrodes, treated with different amounts of the purified antigen UCH37 in PBS buffer.

When NbIII.15, the nanobody used in the current study, binds to its target, an even larger charge injection barrier between redox mediators and the electrode surface should be created and, therefore, a further decrease in current was expected upon antigen binding. A linear decrease in the current recorded with NbIII.15-functionalized graphite felt electrodes with increasing concentrations of UCH37, the target antigen for NbIII.15, was indeed observed. To demonstrate the binding efficiency of the recombinant HaloTag nanobody NbIII.15 towards its target antigen UCH37 and to quantify the efficacy of the encapsulation layer, GF-Nb without any encapsulation was first treated with different buffer solutions containing purified UCH37 at concentrations ranging from 0 to 1.0 *μ*mol. The current signals obtained by performing DPV on unencapsulated GF-Nb samples were significantly noisy and highly variable across multiple electrode samples; therefore, notable antigen binding could not be detected over the noise, even at high antigen concentrations (Fig. 5). The large errors observed for each data point might result from either fiber network variation across different felt samples and/ or loss of the Nb recognition element from the surface of the electrode due to abrasion or mechanical washing during storage and handling. In contrast, electrodes encapsulated with a thin PHEMA layer (denoted as encapsulated GF-Nb) showed minimal baseline signal deviations across multiple samples. The observed current for encapsulated GF-Nb electrodes decreased with increasing UCH37 concentration, as was expected to occur upon antigen binding to the surface-immobilized nanobodies. The average current signal (averaged over three different encapsulated electrodes) displayed Langmuir binding characteristics. The convergent current values generated by different encapsulated GF-Nb samples (fabricated at separate times, using separate functionalization solutions) at the same UCH37 concentration indicates that more robust and reliable working electrodes can be obtained upon encapsulation using piCVD.

To evaluate our graphite-felt biosensors in challenging contexts, we obtained the DPV signal from encapsulated and unencapsulated GF-Nb electrodes when placed into unpurified HEK293 cell lysate (1 mg lysate per 1 ml PBS buffer) containing low concentrations of UCH37 and much larger concentrations of cellular detritus, lipids and various unspecified biomacromolecules (on average, 1 mg of unpurified HEK293 cell lysate will afford approximately 50 picomol of UCH37 after purification).^26^ To probe the specificity of nanobody-modified graphite felt in these challenging conditions, we performed two kinds of control experiments. First, we obtained the DPV signals from encapsulated and unencapsulated samples containing NbIII.15 (which binds UCH37) and a different control nanobody (controlNb) that does not recognize or bind to UCH37. Ideally, electrodes functionalized with the control nanobody should not produce a current change when exposed to UCH37. Second, DPV signals were obtained from NbIII.15 functionalized graphite felt electrodes in a knockout HEK293 cell lysate—UCH37^KO^ lysate —which lacked any UCH37. In this case, NbIII.15 functionalized graphite felt electrodes should not produce a current change when exposed to UCH37^KO^ lysate.

**Figure 6.**
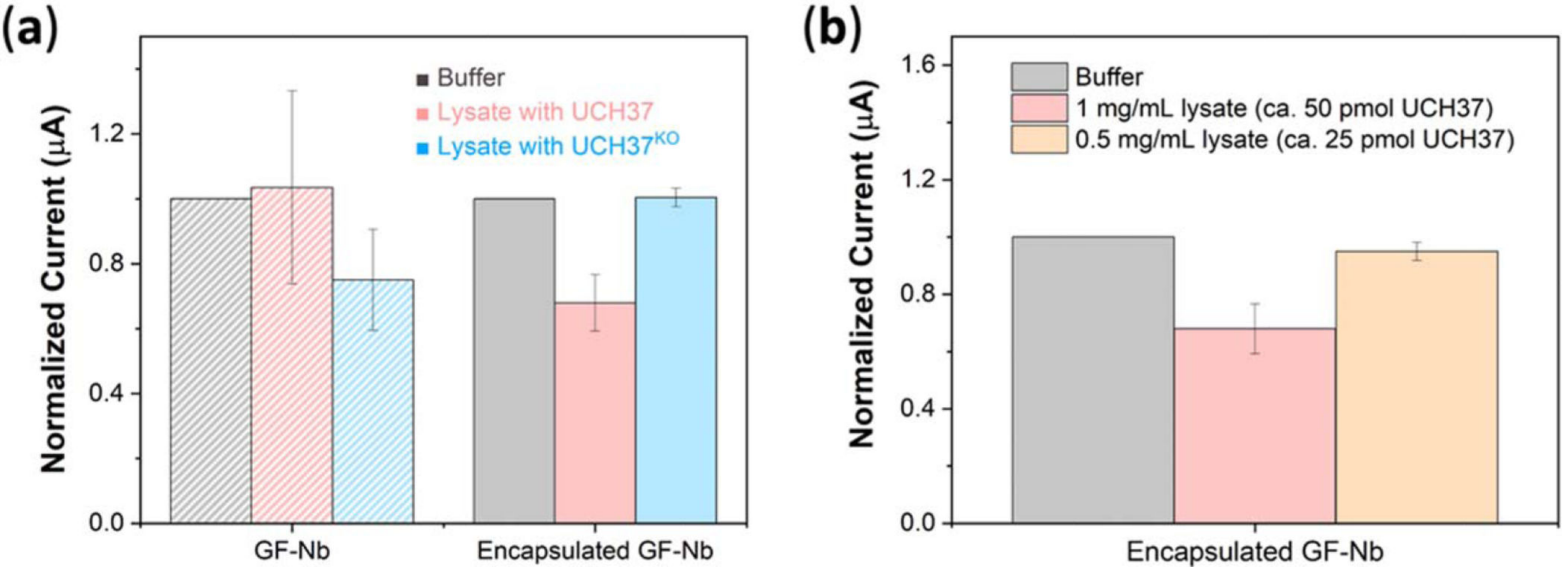
(a) Observed current signals from differential pulse voltammetry performed using bare GF-Nb electrodes and encapsulated GF-Nb electrodes, treated with unpurified HEK293 lysate containing the antigen UCH37 and with a knockout lysate lacking the antigen. (b) Observed current signals from differential pulse voltammetry performed using encapsulated GF-Nb electrodes treated with serially-diluted HEK293 cell lysate to ascertain detection limit (1 mg of unpurified HEK293 cell lysate will afford approximately 50 picomol of UCH37 after purification).

Results for the sensors functionalized with NbIII.15 are provided in Fig. 6 and results from sensors functionalized with the control nanobody are provided in the Supporting Information. When exposed to UCH37 lysate, unencapsulated GF-NbIII.15 electrodes failed to screen out UCH37 from the complex mixture of cellular detritus and also displayed large signal variations across separate electrode samples. Unencapsulated electrodes also afforded false positive signals (with large variations) in control UCH37^KO^ lysate. In contrast, encapsulated GF-NbIII.15 electrodes produced a repeatable current decrease only for UCH37 lysate, and this signal was exactly matched by at least fifteen different electrode samples fabricated at different times over a period of several months. Detailed DPV data are provided in Fig. S4. Moreover, encapsulated GF-NbIII.15 electrodes did not produce any current response in control UCH37^KO^ lysate. Samples containing the control nanobody (that does not bind UCH37 in solution) behaved similarly— unencapsulated samples displayed many false positives and large baseline variations across different electrode samples, whereas encapsulated electrodes containing the control nanobody displayed stable and reproduceable baseline current values across different samples and, also, did not produce any observable current response in UCH37 lysate, as expected (Fig. S5). This strong screen-out capability supported the conclusion that the encapsulation layer created by piCVD provided a barrier that discouraged false positive due to nonspecific binding to the sensor surface while also facilitating transport of target antigens to the underlying recognition elements.

To probe the limit of antigen detection in complex media for the best-performing encapsulated electrodes, unpurified HEK293 cell lysate containing the target antigen UCH37 was serially diluted and the DPV signal for each dilution was measured until a statistically-significant decrease in current could no longer be observed. We defined a statistically-significant response as a current decrease that was equal to or greater than 3× the baseline variation/noise displayed by an encapsulated electrode in plain PBS buffer. Following this procedure, statistically-significant current decreases were observed starting from unpurified cell lysate concentrations of 0.3 mg ml^−1^ and higher (Fig. 6b), while further diluted lysate samples (0.1 mg ml^−1^ ) resulted in noise. In our hands, 1 mg of unpurified HEK293 cell lysate will ultimately yield approximately 50 picomol of UCH37 after purification; therefore, we tentatively concluded that the encapsulated graphite felt electrode displayed a target antigen detection limit of 25–30 picomol in lysate. We expect that this limit of detection can be further reduced by increasing the loading of the recombinant nanobody recognition element on the graphite felt electrode.

## Conclusions

In this study, we report a general strategy to fabricate robust, sensitive, and selective electrochemical sensors incorporating nanobodies for directly detecting target analytes in complex, heterogeneous media, such as cell lysate, without added purification or preconcentration steps. Graphite felt serves as a readily-functionalizable substrate that can be covalently decorated with recombinant HaloTag-modified nanobodies. Subsequent encapsulation with a thin layer of a hydrogel, poly(2-hydroxyethyl methacrylate) (PHEMA), using a photoinitiated chemical vapor deposition process affords uniformly encapsulated electrodes that directly display a decrease in current upon antigen binding, without the need for added redox mediators.

Differential pulse voltammetry affords clear and consistent decreases in electrode current across multiple encapsulated electrode samples for specific antigen concentrations. The change in observed current vs increasing antigen concentration follows Langmuir binding characteristics, as expected. Importantly, selective and repeatable target binding in unpurified cell lysate is only demonstrated by the encapsulated electrode, with an antigen detection limit of ca. 30 pmol, whereas bare electrodes lacking a hydrogel encapsulation produce highly-variable baseline current values and numerous false positive signals in control experiments.

The three-dimensional network structure of graphite felt provides a large surface area for immobilization of recognition elements (nanobodies in this study) and facilitated mass transport for efficient target-binding (UCH37 in this study). However, since electrochemical measurements are highly sensitive, the disordered fibrous network of graphite felt can also lead to large background signals and unwanted noise during data acquisition. In the current study, controlled electrode encapsulations were conducted to largely eliminate such issues, but further sensor engineering is still required to increase the signal-to-noise ratio of graphite felt-based working electrodes to a point where their performance matches those of conventional metal-based electrodes.

While the nanobody/antigen combination used for these studies, NbIII.15/UCH37, was chosen simply because of its availability in our labs, the procedures we report herein can be easily applied to any known nanobody-antigen combination or used to develop nanobody-based electrochemical biosensors for any biomolecule or target of interest.^32^

## Supplementary Material

Supporting Info
