# Supplementary material for: A Strategy for Accessing Nanobody-Based Electrochemical Sensors for Analyte Detection in Complex Media
Source: ECS Sens Plus. Author manuscript; Available in PMC 2022 Nov 4. (PMC9635334; doi:10.1149/2754-2726/ac5b2e)
Supplement: Supporting Info [file NIHMS1810208-supplement-Supporting_Info.docx]

***Supplementary Material***

# Supplementary Figures


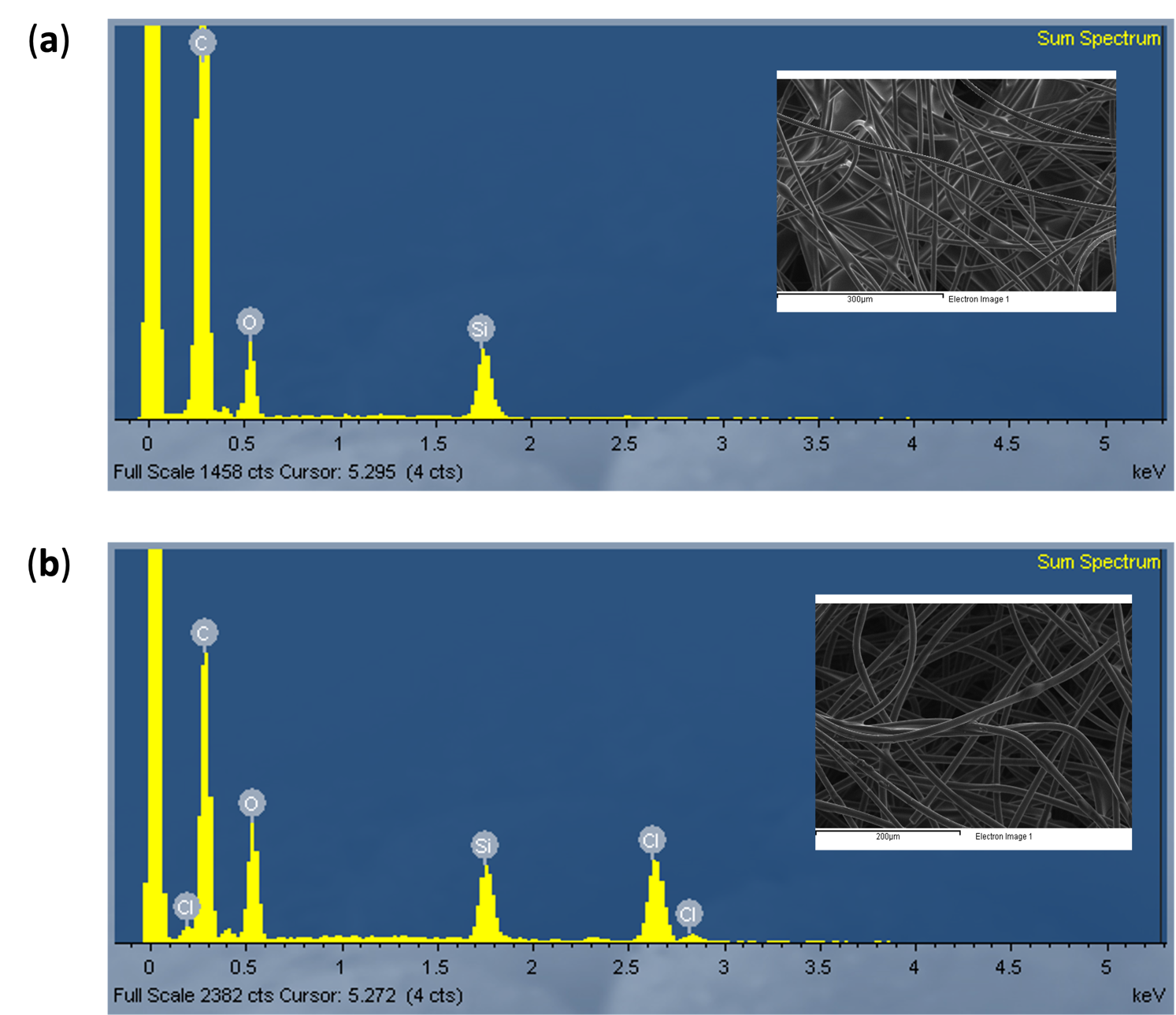


**Supplementary Figure 1.** EDX elemental analysis of (a) GF treated with APTMS (GF-Si) and (b) GF treated with APTMS followed by CHA (GF-Cl)


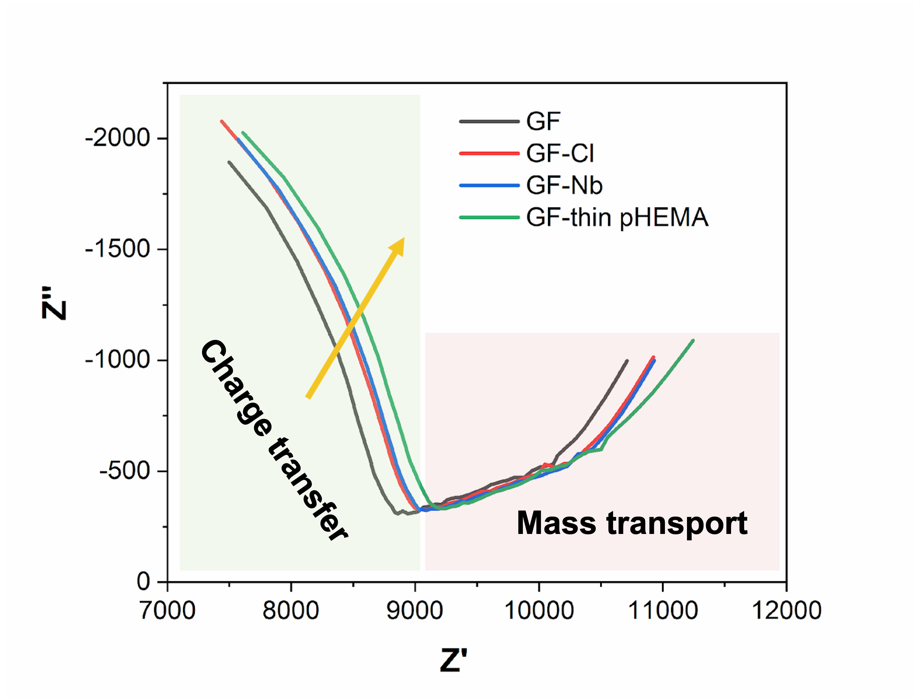


**Supplementary Figure 2.** Impedance of functionalized GF electrode


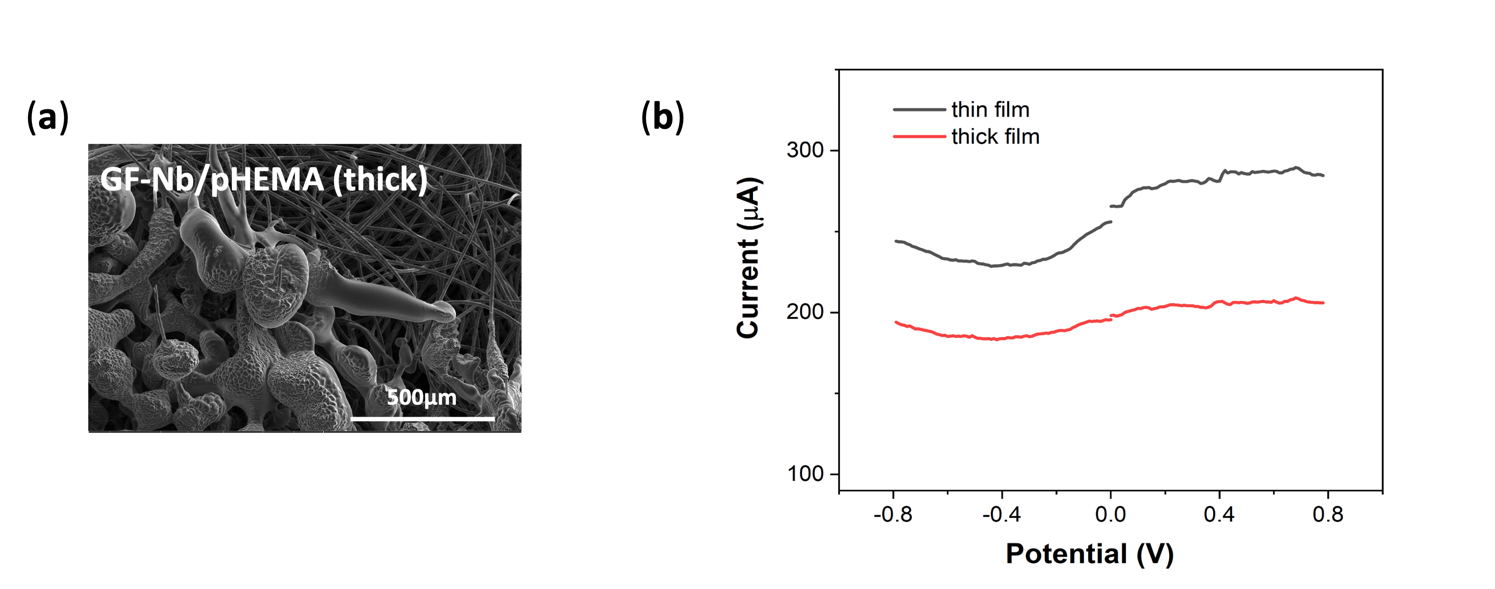


**Supplementary Figure 3.** (a) SEM of GF-Nb/pHEMA (thick), (b) DPV measurements of GF-Nb with thin and thick pHEMA film


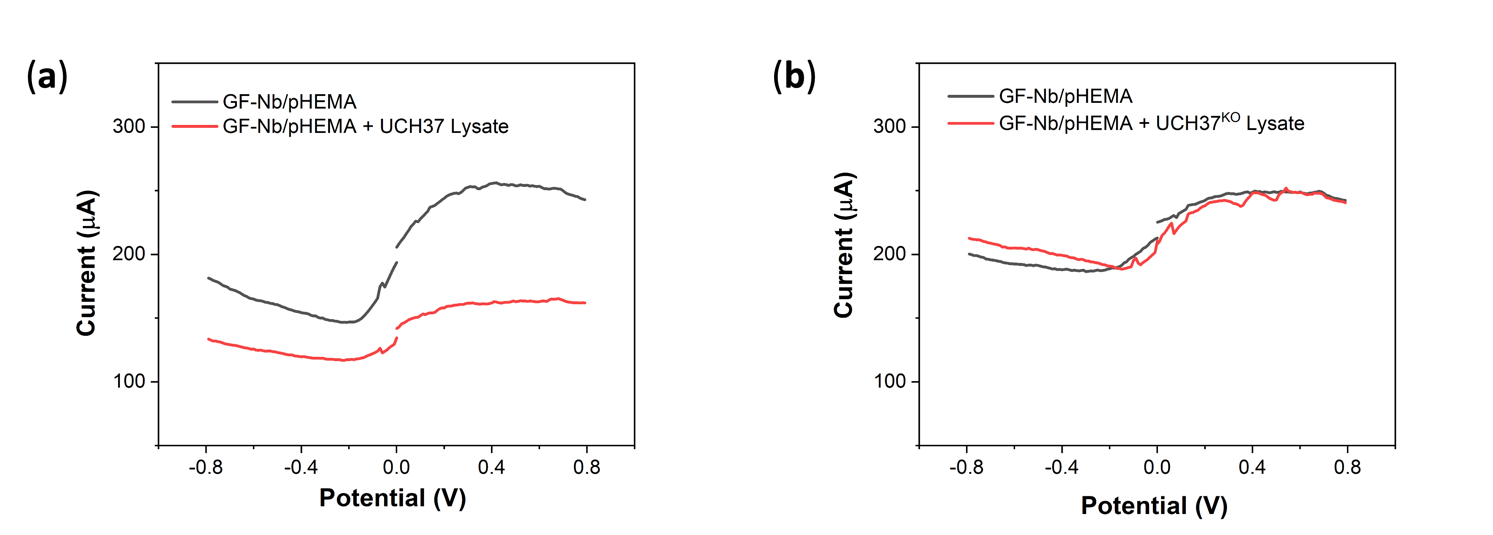


**Supplementary Figure 4.** DPV data of (a) GF-Nb/pHEMA sensors towards to UCH37 Lysate

and (b) GF-Nb/pHEMA sensors towards to UCH37^KO^ Lysate

**
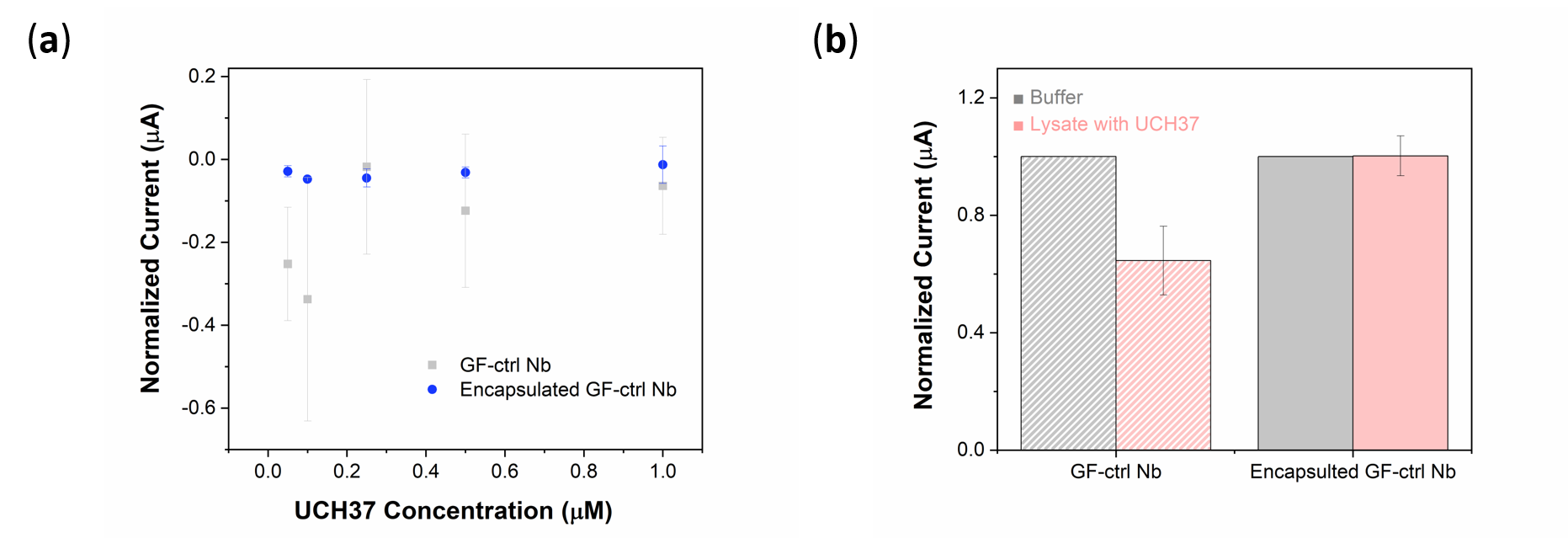
**

**Supplementary Figure 5.** (a)Responses of GF-ctrl Nb and GF-ctrl Nb/pHEMA to pure UCH37 with different concentration and (b)GF-ctrl Nb and GF-ctrl Nb/pHEMA treated with lysate
